# Inhibition of SARS-CoV-2 pseudovirus invasion by ACE2 protecting and Spike neutralizing peptides: An alternative approach to COVID19 prevention and therapy

**DOI:** 10.7150/ijbs.61476

**Published:** 2021-07-13

**Authors:** Jiang Chen, Song Li, Zhifeng Lei, Qinmin Tang, Ling Mo, Xing Zhao, Feifei Xie, Dan Zi, Jun Tan

**Affiliations:** 1 Key Laboratory of Endemic and Ethnic Diseases, Laboratory of Molecular Biology, Ministry of Education, Guizhou Medical University, Guiyang 550004, China; 2 The First Affiliated Hospital of Dalian Medical University, Dalian 116021, China; 3 Key Laboratory of Adult Stem Cell Transformation Research, Chinese Academy of Medical Sciences/Stem Cell and Tissue Engineering Research Center, Guizhou Medical University, Guiyang 550004, China; 4 Anyu Biopharmaceutical (Hangzhou) Co., Ltd. 9F, Building I, No. 265, Chengrui Street, Qiantang New District, Hangzhou 310018, China; 5 Department of Obstetrics and Gynecology, Affiliated Hospital of Guizhou Medical University, Guiyang 550004, China

**Keywords:** SARS-CoV-2, COVID-19, peptide, ACE2, spike protein, cocktail therapy

## Abstract

SARS-CoV-2 invades host cells mainly through the interaction of its spike-protein with host cell membrane ACE2. Various antibodies targeting S-protein have been developed to combat COVID-19 pandemic; however, the potential risk of antibody-dependent enhancement and novel spike mutants-induced neutralization loss or antibody resistance still remain. Alternative preventative agents or therapeutics are still urgently needed. In this study, we designed series of peptides with either ACE2 protecting or Spike-protein neutralizing activities. Molecular docking predicted that, among these peptides, ACE2 protecting peptide AYp28 and Spike-protein neutralizing peptide AYn1 showed strongest intermolecular interaction to ACE2 and Spike-protein, respectively, which were further confirmed by both cell- and non-cell-based in vitro assays. In addition, both peptides inhibited the invasion of pseudotype SARS-CoV-2 into HEK293T/hACE2 cells, either alone or in combination. Moreover, the intranasal administration of AYp28 could partially block pseudovirus invasion in hACE2 transgenic mice. Much more importantly, no significant toxicity was observed in peptides-treated cells. AYp28 showed no impacts on ACE2 function. Taken together, the data from our present study predicted promising preventative and therapeutic values of peptides against COVID-19, and may prove the concept that cocktail containing ACE2 protecting peptides and spike neutralizing peptides could serve as a safe and effective approach for SARS-CoV-2 prevention and therapy.

## Introduction

The severe acute respiratory syndrome coronavirus 2 (SARS-CoV-2) causes coronavirus disease 2019 (COVID-19) with rapid global socioeconomic disruptions and disease burden to healthcare 1. As of 8 March 2021, there have been 122,992,844 confirmed cases of COVID-19, including 2,711,071 deaths (2.2%) (World Health Organization. https://covid19.who.int/). SARS-CoV-2 belongs to the *Sarbecovirus subgenus* (*genus Betacoronavirus*, family *Coronaviridae*) 2 together with SARS-CoV that emerged in 2002 causing more than 8000 infections with a lethality of 10% 3. Both viruses crossed species barriers from an animal reservoir and can cause a life-threatening respiratory illness in humans. Despite several types of vaccines or antibodies have been developed and authorized around the globe, to date, only limited prophylactics or therapeutics, such as dexamethasone and budesonid, are clinically available for the highly pathogenic COVID-19 infections in human, highlighting the urgent need for therapeutics discovery.

SARS-CoV-2 is RNA virus containing four structural components including spike glycoprotein (S-protein), membrane, envelope, and nucleocapsid. SARS-CoV-2 attaches to the host cells with the aid of the S-protein present on its envelope. Coronavirus S-protein is composed of two subunits, S1 and S2. The S1 subunit contains the receptor binding domain (RBD), which enables the virus to attach to the angiotensin-converting enzyme 2 (ACE2) receptor on host cells 4. The S2 subunit is responsible for the fusion of virus and the host cell membrane, with the aid of transmembrane serine protease 2 and furin, which cleave the full-length S glycoprotein at S1/S2 or S2' site, respectively 5. S-protein is currently the most important target for not only candidate vaccines but also other potential antivirals, as it mediates the virus attachment and invasion into host cells and is the target of neutralizing antibody responses 6-9. However, safety concerns still remain due to potential risk of antibody-dependent enhancement (ADE) and novel spike mutants-induced neutralization loss or antibody resistance 10-13.

ACE2, an enzyme located on the outer surface of a wide variety of cells, is the primary host cell target with which the S-protein of SARS-CoV-2 associates 14, 15. Despite the S-protein-targeted neutralizing antibodies and nanobodies 16, 17, ACE2-targeted agents, including pharmacological small molecule compounds, peptides and proteins, have been proposed to block the interactions of SARS-CoV-2 with ACE2 18, 19. Recombinant SARS-CoV-2 RBD protein has also been reported to block the binding and attachment of SARS-CoV-2 to ACE2, thus inhibit SARS-CoV-2 invasion into host cells 20. However, it is worth noting that SARS-CoV spike injection in mice worsened lung injury, which was attenuated by blocking the renin-angiotensin pathway and depended on ACE2 expression 21. Therefore, for SARS-CoV or SARS-CoV-2 pathogenesis, ACE2 serves not only the entry receptor of the virus but also the protector against lung injury. Protective agents which specifically block ACE2 without inhibitions on ACE2 activities should be a safe and effective approach to combat SARS-CoV-2 infection.

In our present study, we designed series of peptides based on the sequence of S-protein RBD region of SARS-COV-2 or SARS-COV and the sequence of hACE2 (**Fig. S1a and S1b**). Molecular docking predicted that, among these peptides, ACE2 protecting peptide AYp28 and S-protein neutralizing peptide AYn1 showed strongest intramolecular interactions to their targeting molecules, which were further confirmed by both cell- and non-cell-based *in vitro* assays. In addition, both peptides inhibited the invasion of pseudotype SARS-CoV-2 into human ACE2 over-expressing HEK293T cells (HEK293T/hACE2), either alone or in combination. Moreover, the intranasal inoculation of AYp28 peptide partially prevented and inhibited pseudovirus infection in hACE2 transgenic mice. Much more importantly, no significant toxicity was observed in peptides-treated cells and mice. ACE2 protecting peptide AYp28 showed no impacts on ACE2 function of converting angiotensin II into angiotensin 1-7. Although AYn1 peptide failed to prevent pseudovirus infection in vivo, the data from our present study predicted promising preventative and therapeutic values of peptides against COVID-19, and may prove the concept that cocktail containing ACE2 protecting peptides and spike neutralizing peptides could serve as a safe and effective approach for SARS-CoV-2 prevention and therapy.

## Results and Discussion

**Computational simulation of peptides-proteins interactions.** ACE2 protecting peptides (AYp1-28) were designed and modified based on the sequence of S-protein RBD region of SARS-COV-2 (VEGFNCYFPLQS) or SARS-COV (YKYRYLRHGKLR) as shown in **Fig. S1a**. S-protein neutralizing peptides (AYn1-14) were designed and modified according to the sequence of hACE2 (DKFNHEAEDLFY, MYPLQEIQNLTV, and GKGDER, **Fig. S1b**). Peptide-protein interactions were computationally simulated using Rosetta FlexPepDock web server (http://flexpepdock.furmanlab.cs.huji.ac.il/). Peptides sequences, their docking proteins and binding parameters were summarized in **Table 1** and **Table 2**, in which those peptides with stronger binding affinities were highlighted in *italic*. Our data revealed that, among 21 tested ACE2 protecting peptides, AYp28 showed a larger attachment surface with ACE2 protein (yellow area in **Fig. S2**) and better binding affinity to ACE2 protein, as evidenced by the lowest dG_cross value and relatively higher dSASA_int and sc_value, together with a lowest I_sc value (**Table 1**). Among 14 tested S-protein neutralizing peptides, AYn1 presented the largest binding surface (yellow area in **Fig. S3**) and a better binding affinity to S-protein (**Table 2**). Interestingly, our data suggested that modification by 6-lysine could enhance the binding affinity of these peptides to their respectively targeting proteins. For example, the 6K-modified AYp28 peptide (KKKKKKVEGFNCYFPLQS) showed a stronger binding affinity to ACE2 than its template peptide AYp27 (VEGFNCYFPLQS) did. The dG_cross, dSASA_int and sc_values of AYp28 and AYp27 were -34.4496 *vs* -31.5179, 1531.23655 *vs* 1121.02775, and 0.56795 *vs* 0.65705, respectively; Similarly, the 6K-modified AYn1 peptide (KKKKKKDKFNHEAEDLFY) showed stronger binding affinities to S-protein than non-modified AYn8. The dG_cross, dSASA_int and sc_values of AYp28 and AYp27 were -26.67675 *vs* -25.00335, 1299.1106 *vs* 989.77225, and 0.5374* vs* 0.5769, respectively. Considering their better scores in molecular docking, AYp28 and AYn1 were selected for further cell- or non-cell-based biological function analysis and pseudovirus invasion tests both *in vitro* and *in vivo*.

**Figure 1:**
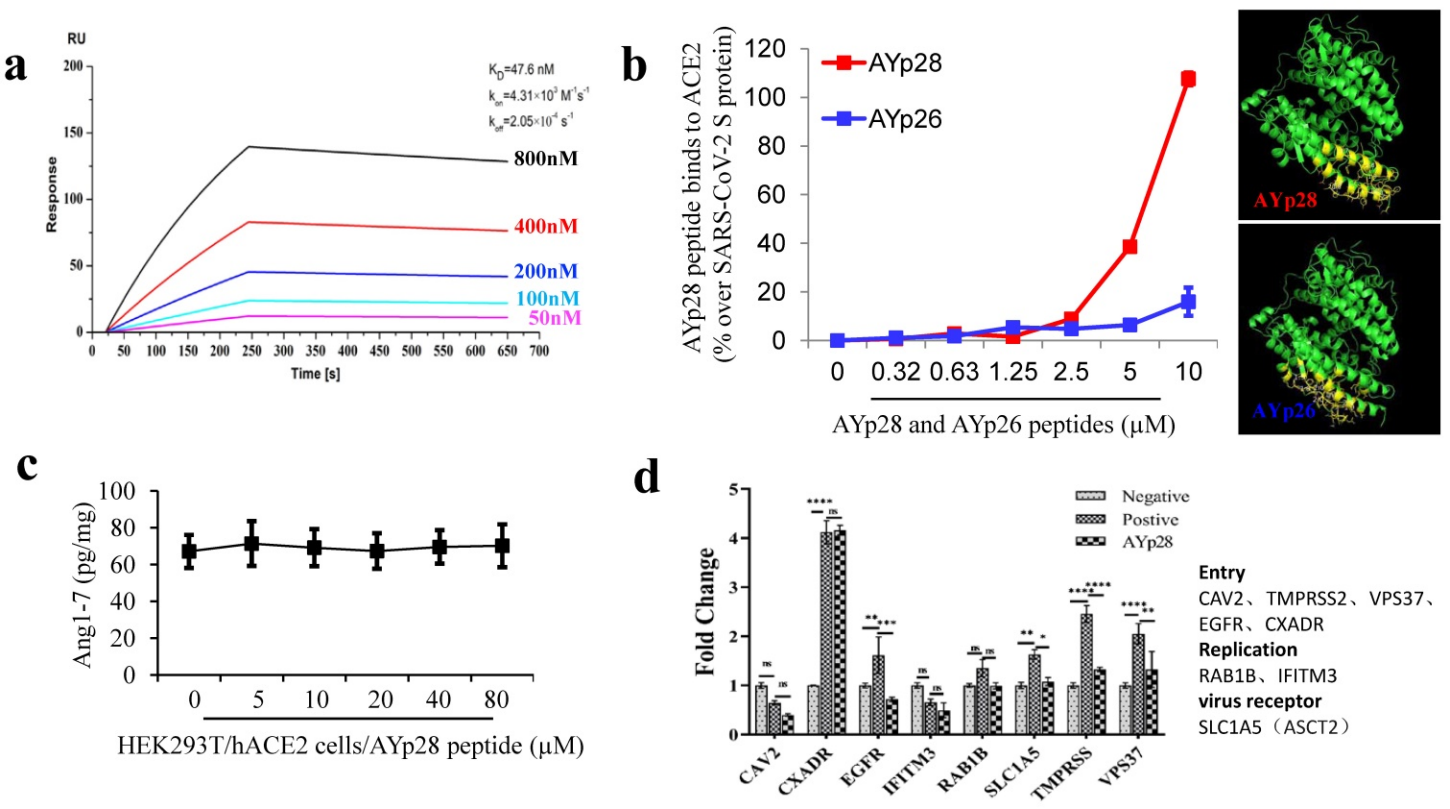
** ACE2 protecting peptide (AYp28) highly binds to ACE2 protein without ACE2 function alteration.** (**a**) ACE2, captured on COOH chip, can bind to AYp28 with an affinity constant of 47.6 nM as determined by LSPR assay. (**b**) As in cell-free system confirmation, a 96-well plate was coated with either AYp28 (V475-S486 of SARS-CoV-2 S-protein) or AYp26 peptide (Y438-R449 of SARS-CoV S-protein) at 0 to 10 µM for overnight at 4 °C, followed by adding human recombinant ACE2 protein with His-tag at 100 ng/mL for 2 hours at room temperature. Routinely, the binding affinity was analyzed by His-tag enzyme-linked immunosorbent assay (ELISA). As a positive control, the paralleled 96-well plate was also coated with SARS-CoV-2 S protein at 10 ng/mL. ELISA results are representative of three independent experiments, with each condition duplicated and presented as the mean ± SD of AYp28 or AYp26 peptide binding affinity [OD reading (% over SARS-CoV-2 S-protein)]. (**c**) As a functional confirmation, HEK293T/hACE2 cells were plated in 6-well plates for 20 hours and treated with AYp28 at 0-80 µM for 1 hour, followed by the addition of Ang II at 10 nM for 30 minutes, and then ACE2 activity in the conditioned media by Ang1-7 ELISA. ELISA results are representative of two independent experiments, with each condition triplicated and presented as the mean ± SD of ACE2 activity [Ang1-7 (pg/mg total intracellular protein)]. (**d**) HEK293T/hACE2 cells were pre-treated with AYp28 peptide at 20 µM for 30 min and then challenged with pseudovirus with the S-protein at 10^10^/mL for 2 hours at 37℃, followed by analysis of mRNA profile by a real-time PCR.

**ACE2 protecting peptide AYp28 highly binds to ACE2 without altering ACE2 function.** In order to further confirm the binding ability of ACE2 protecting peptide AYp28 to ACE2, localized surface plasmon resonance (LSPR) assay and non-cell based peptide-protein binding assay were performed. The data of LSPR assay clearly revealed a strong interaction between AYp28 and ACE2, with an affinity constant of KD=47.6 nM (**Fig. 1a**). Consistently, non-cell based peptide-protein binding assay also confirmed strong interactions of AYp28 with ACE2 (**Fig. 1b**). Interestingly, our data further indicated that, at 5 to 10 µM concentration range, the SARS-CoV-2 derived AYp28 showed much stronger binding affinity to ACE2 than SARS-CoV derived peptide AYp26 (**Fig. 1b**), supporting the stronger infectivity of SARS-CoV-2 than that of SARS-CoV.

Moreover, biotin-labeled AYp28 peptide was incubated with HEK293T/hAEC2 cells and observed by confocal microscopy. Consistent with those non-cell based assays, clear colocalizations of AYp28 with cell membrane ACE2 were found (**Fig. S4**). Worth noting, while showing strong binding ability to ACE2, AYp28 peptide did not inhibit ACE2 function and thus presented a good safety profile. As shown in **Fig. 1c**, in HEK293T/hAEC2 cells, the AYp28-bound ACE2 can still convert Ang II into Ang 1-7, indicating an intact enzyme activity. Moreover, the mRNA levels of various proteins involved in virus membrane transport or virus entry were further detected to evaluate potential impacts of peptides. As shown in **Fig. 1d**, after pseudovirus infection, compare with those pseudovirus-infected cells without AYp28 treatment, the levels of TMPRSS, VPS37 and EGFR, which responsible for the virus entries into host cells, were significantly decreased in AYp28 treated cells, indicating an impaired virus entrance process. In addition, no significant changes (ns) were observed on the expression level of RAB1B and IFITM3, two proteins responsible to virus replication. Moreover, AYp28 treatment also showed no impact on the expression level of another cell membrane protein SLC1A5, suggesting the relatively high specificity of the impact of AYp28 on ACE2 (**Fig. 1d**).

Additionally, the data from MTT assay further indicated that AYp28 under 31.25 μM concentrations showed no significant cytotoxicity in HEK293T/hACE2 cells after 48 hours coincubation (**Fig. S5a and S5b**). The lower cytotoxicity together with the intact ACE2 enzyme activity after peptide exposure in vitro may predict a good safety profile of AYp28 for future in vivo application.

**Figure 2:**
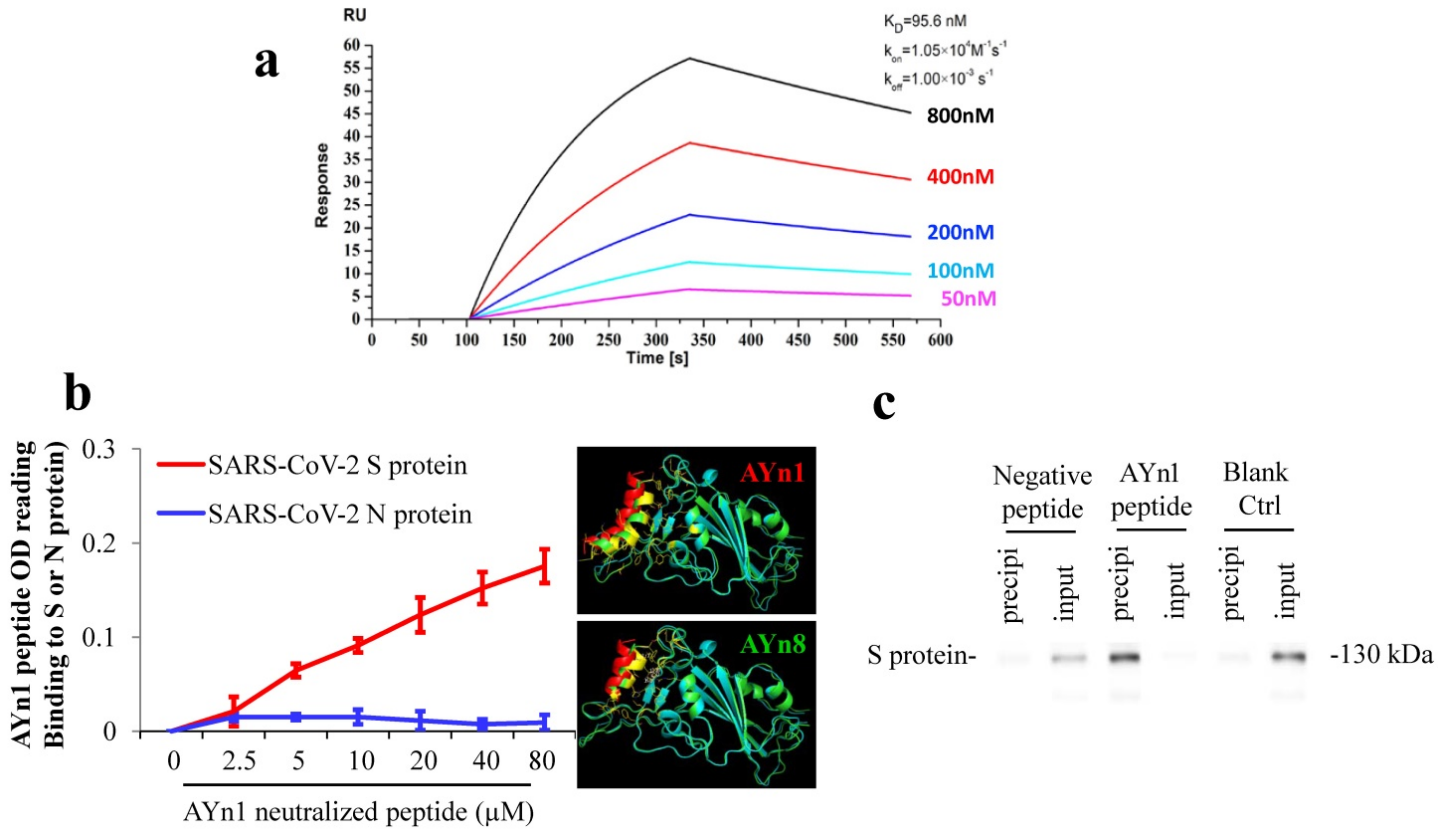
** SARS-CoV-2 S-protein neutralizing peptide (AYn1) specifically binds to S-protein.** (a) S-protein RBD, captured on COOH chip, can bind to AYn1 with an affinity constant of 95.6 nM as determined by LSPR assay; (**b**) As in cell-free confirmation, a 96-well plate was coated with SARS-CoV-2 S or N protein at 10 ng/mL for overnight at 4℃, followed by adding AYn1 and control peptides with His-tag at 0 to 80 µM respectively for 2 hours at room temperature. Routinely, the binding affinity was analyzed by His-tag ELISA. (**c**) The peptide-protein interactions were further conformed by pull-down experiment. Biotinylated AYn1 peptides (100 μL, 40 μM) was mixed with Dynabeads M-280 streptavidin at room temperature for 15 minutes. AYn1 peptide without biotin or PBST buffer was mixed with beads as negative control or blank control. SARS-CoV-2 S-protein (1 μL, 0.25 μg/μL) was added to the beads and mixed for 20 minutes. The supernatant and heat elution from the beads were loaded on SDS-PAGE gel and determined using Anti-SARS-S1 rabbit polyclonal antibody. While no protein signal was detected in negative and blank control lanes, a strong protein-peptide interaction was observed in AYn1-immobilized condition.

**SARS-CoV-2 S-protein neutralizing peptide AYn1 specifically binds to S-protein.** We further tested the specific binding of S-protein neutralizing peptide AYn1 to virus S-protein. As expected, LSPR assay confirmed a strong interaction between AYn1 and S-protein with an affinity constant of KD=95.6 nM (**Fig. 2a**). In addition, non-cell based peptide-protein binding assay also revealed a strong interaction between AYn1 and S-protein (**Fig. 2b**), consistent with the results of molecular docking and LSPR. Importantly, our data further indicated that, at 0 to 80 µM concentration range, AYn1 peptide showed no affinity to virus nucleocapsid protein (N-protein) (**Fig. 2b**), suggesting a specific binding of AYn1 to SARS-CoV-2 S-protein. This specific binding was further confirmed by pull-down analysis. While no protein band was detected in negative and blank control lanes, a strong protein-peptide interaction was observed in AYn1-immobilized condition (**Fig. 2c**). Moreover, similar to AYp28, MTT assay indicated no significant cytotoxicity of AYn1 (<31.25 μM concentrations) in HEK293T/hACE2 cells (**Fig. S5c and S5d**).

**Figure 3:**
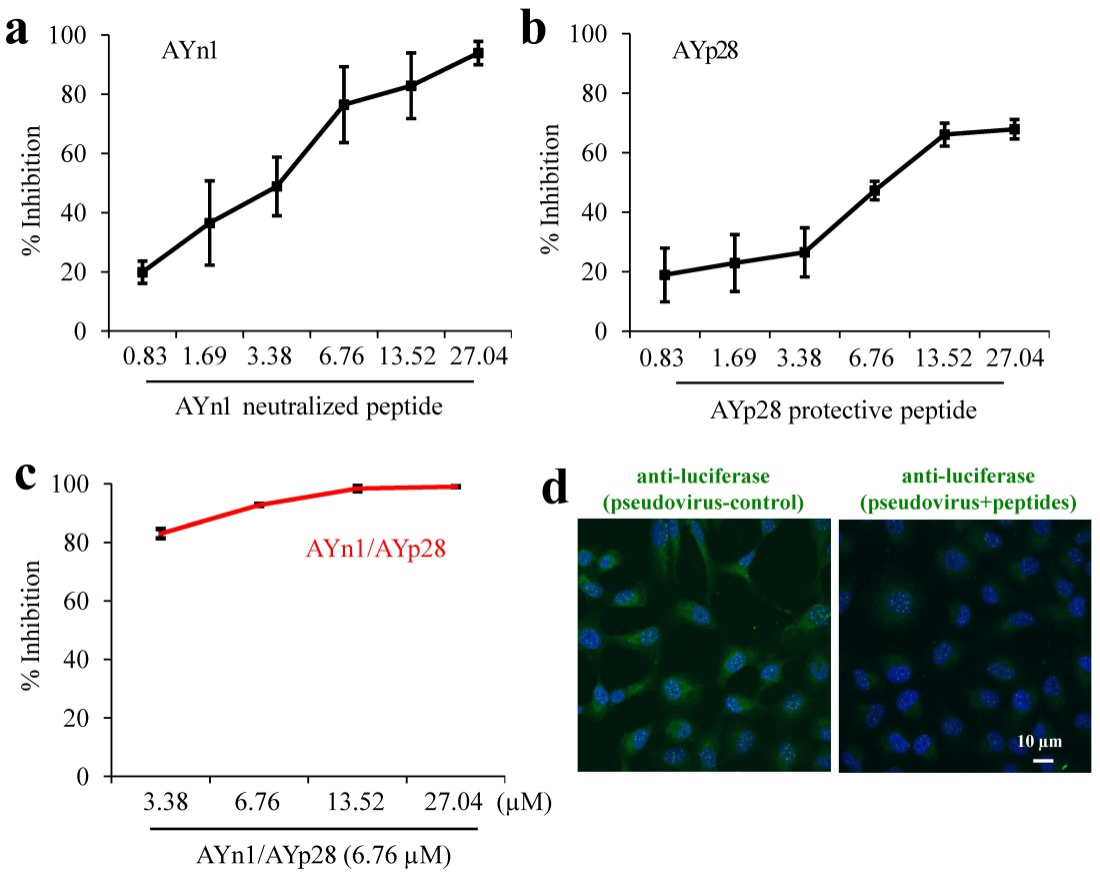
** Synergistic inhibition of AYn1 and AYp28 peptides on pseudovirus invasion into HEK293T/hACE2 cells.** (**a**) Pseudovirus at 10^10^/mL were incubated with AYn1 at 0 to 27.04 µM for 2 hours at 37ºC, followed by further respectively incubating with HEK293T/hACE2 cells in 96-well plate for 48-72 hours at 37ºC. (**b**) HEK293T/hACE2 cells at 30,000 cells/well were plated in 96-well plates and incubated with AYp28 at 0 to 27.04 µM at 37ºC for 2 hours, followed by adding the pseudovirus at 10^10^/mL for 48-72 hours at 37ºC. (**c**) For synergistic assay, the neutralized pseudovirus (preincubated with 0 to 27.04 µM of S-protein neutralizing peptide AYn1 for 2 hours at 37ºC) were added into HEK293T/hACE2 cells pretreated with 5 µM ACE2 protecting peptide AYp28, and further incubated for 48-72 hours at 37°C. Luciferase activity was measured. The results are representative of three independent experiments, with each condition triplicated and presented as the mean ± SD of inhibition of the pseudovirus. (**d**) Inhibition effect of peptidic cocktail on pseudovirus invasion in HEK293T/hACE2 cells was further confirmed by confocal microscopy. The fluorescence of pseudovirus (green), peptides (red) and nuclei (blue) were observed. Scale bar = 10 µm.

**Combination treatment of AYn1 and AYp28 peptides inhibits pseudovirus invasion into HEK293T/hACE2 cells.** As shown in **Fig. 3**, both AYn1 and AYp28 could block the invasion of pseudo-SARS-CoV-2 virus into HEK293T/hACE2 cells in a dose dependent manner, with IC_50_ values at 4.9 µM for AYn1 (**Fig. 3a**) and 14.6 µM for AYp28 (**Fig. 3b**). In addition, the combination of 6.76 µM AYp28 with various concentrations of AYn1 showed a synergistic effect, as evidenced by the elevated neutralization percentage (especially at 3.38 µM,** Fig. 3c**) and the decreased IC_50_ value of 2.5 µM. This inhibition effect of peptidic cocktail on pseudovirus invasion in HEK293T/hACE2 cells can be further confirmed by confocal microscopy observation. As shown in **Fig. 3d (left Panel)**, pseudovirus-infected cells showed a bright fluorescence (green). In contrast, peptides cocktail treatment can significantly weaken pseudovirus invasion, as evidenced by the decreased green fluorescence intensity (**Fig. 3d, right panel**).

**Figure 4:**
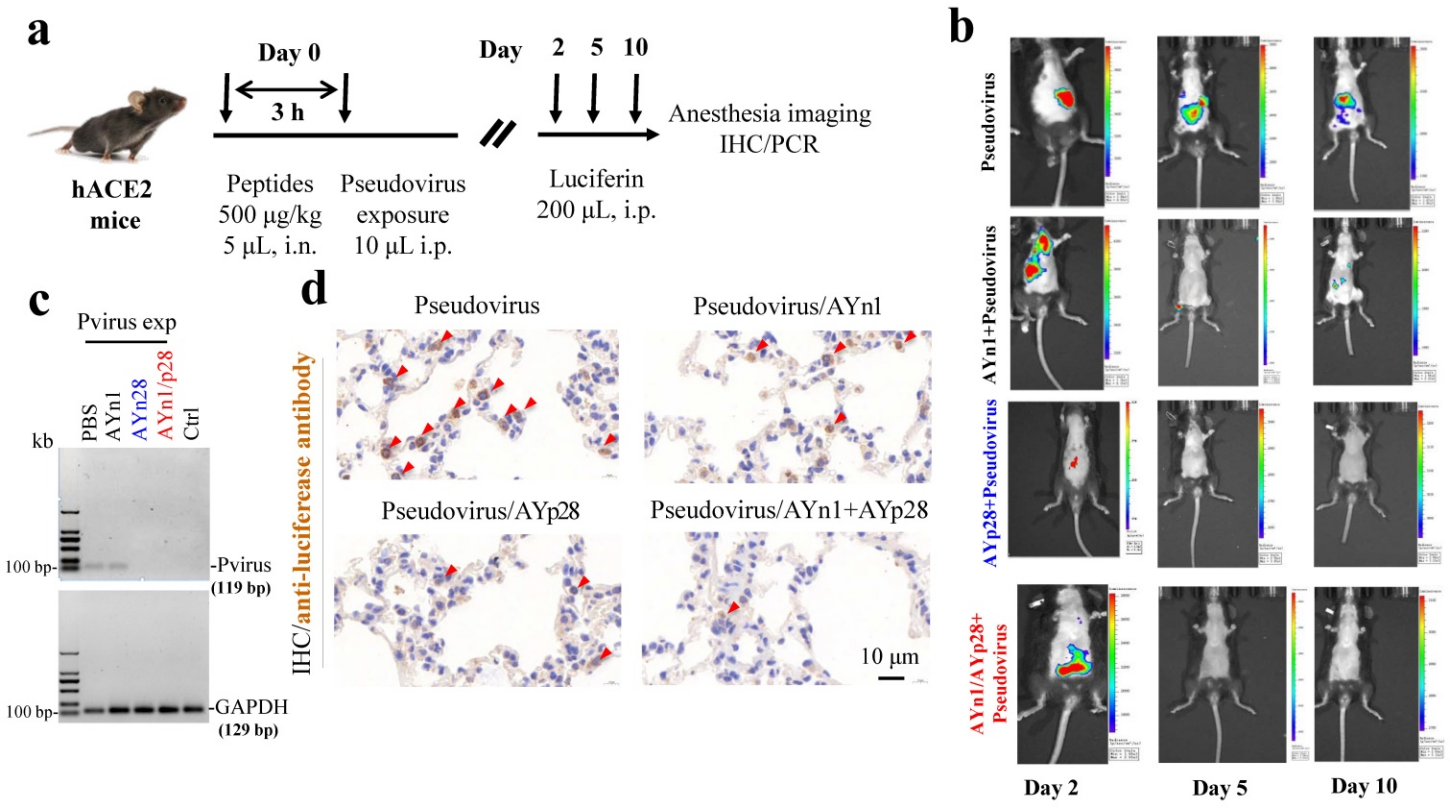
** Inhibition of AYn1 and AYp28 peptides on pseudovirus invasion in hACE2 mice.** (**a**) Schematic illustration of experimental procedure. Pseudovirus containing luciferase reporter gene was intranasally (i.n.) administered to hACE2 mice 3 hours after peptides treatment (500 μg/kg, 5 μL). After pseudovirus exposure, animals were intraperitoneally (i.p) injected with Luciferin (200 μL) and subjected to imaging at day 2, 5 and 10. After final imaging observation, all animals were sacrificed for IHC staining and RT-PCR. **(b)** The dynamic changes of pseudovirus invasion in mice were observed by using IVIS Lumina III system. The luciferin luminance can be ameliorated by AYp28, AYn1 or AYn1/AYp28 treatment. (**c**) Ten days post pseudovirus exposure (Pvirus exp), the pseudovirus content in lungs was quantified by using RT-PCR, as indicated by the 119 bp S-protein band. Pseudovirus can be detected in PBS-treated group, which can be diminished by treatment with AYp28 or AYp28/AYn1. AYn1 treatment alone showed a relative weak therapeutic effect. **(d)** Pseudovirus locations in lungs 10 days after pseudovirus exposure were further confirmed by IHC staining using anti-luciferease antibody. Consistent with the data of RT-PCR, clearly positive staining can be observed, which can be significantly ameliorated by pre-treatment with AYp28, AYn1 or AYp28/AYn1 combination.

**Preventative effects of AYp28 peptides on pseudovirus invasion in hACE2 mice.** To further determine the in vivo preventative of AYn1, AYp28 and their combination on the pseudovirus invasion, hACE2 transgenic mice were intranasally dripped with biotin-labeled peptides and then intraperitoneally exposed to SARS-CoV-2 pseudovirus expressing a luciferase reporter gene (**Fig. 4a**). The pseudovirus infection and potential impacts of peptides treatment were monitored by luceferin imaging using IVIS Lumina III system. The distributions of pseudovirus and peptides were further detected by PCR and/or IHC staining. While no luciferin signal was observed in PBS and luciferin control groups (**Fig. S6**), the pseudovirus-exposed control mice showed a progressive and stable pseudovirus infection, as evidenced by the strong luciferin signal at day 2, 5 and 10 post pseudovirus exposure, although the signal density was gradually declined on 5 and 10 days (**Fig. 4b**). Compare with pseudovirus exposed control animals, animals pretreated with AYp28 at 500 μg/kg showed a significant reduction of the virus load as evidenced by the decreased luciferin signal density on day 2. Although not a complete blockage, this reduction may predict a promising way to prevent COVID19 infection *in vivo*. In contrast to AYp28, AYn1 treatment showed a relatively weaker impact on pseudovirus infection. Moreover, the combined exposure of AYp28 and AYn1 showed no preventative effect on pseudovirus infection. This might be due to the direct interactions between AYp28 and AYn1 in nasal cavity and the relatively lower concentrations. Further experiments are required to explore the detailed dose-response relationship of these peptides in vivo.

**Figure 5:**
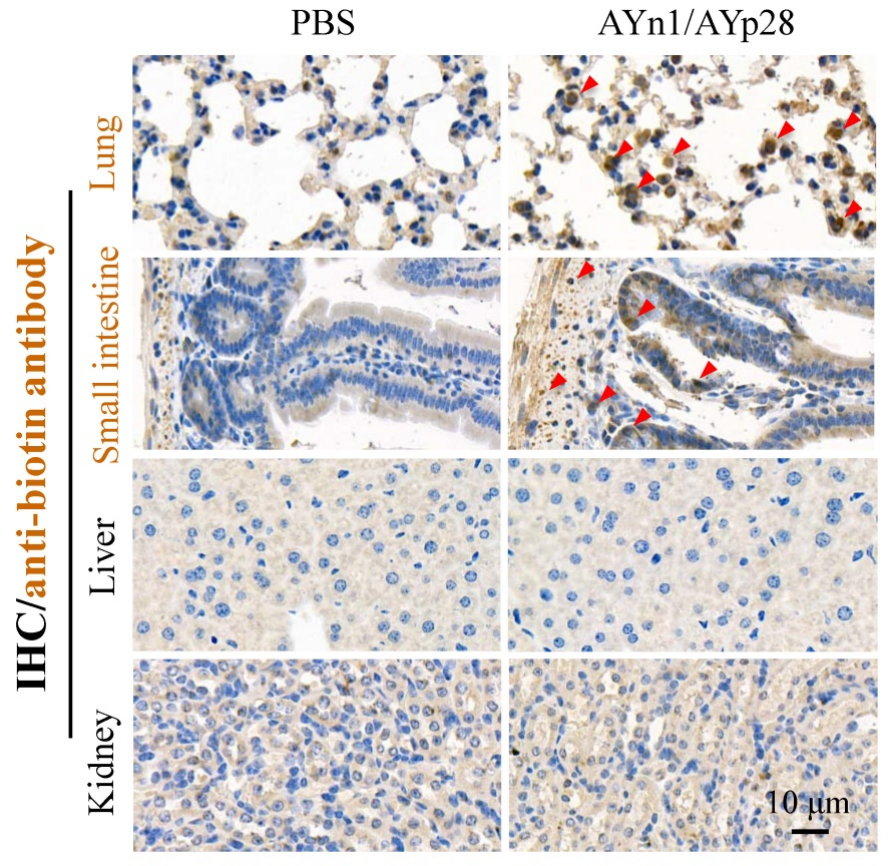
** AYn1/AYp28 peptides mainly targets lung and small intestine tissues hACE2 mice after intranasal administration.** Ten days after intranasal administration of biotin-labeled peptides, the distribution of AYn1 and AYp28 peptides were examined by IHC using anti-biotin antibody. Our data indicated that 10 days after intranasal administration, peptides preferentially distributed in the lungs and intestine, but not in the kidneys and livers.

Interestingly, at day 10 post pseudovirus exposure, although no luciferin signal was observed in the thorax of pseudovirus-exposed control mice, the PCR and IHC staining still found that pseudovirus can be distributed there, as evidenced by 119 bp bands (**Fig. 4c**) and intense positive IHC staining of luciferase (**Fig. 4d**). This infection was also inhibited by AYp28 alone and AYp28/AYn1 combined treatment, as evidenced by the diminished 119bp bands in **Fig. 4c** and the decreased positive luciferase staining in **Fig. 4d**. Moreover, the IHC staining also confirmed that 10 days after treatment of biotin-labeled peptides, the peptides can spread from nasal cavity to the lungs and small intestine, as shown by the positive IHC staining of biotin, but not in livers and kidneys (**Fig. 5**).

The enormous burden imposed by the COVID-19 pandemic on our society and the profound consequences for the personal, social, health-related and economic aspects of our daily lives has triggered a race towards the discovery and development of therapies that act against SARS-CoV-2. The initial step for a rapid therapeutics discovery is to search for potential candidates from existing drugs such as hydroxychloroquine, chloroquine, remdesivir, and lopinavir etc. for the treatment of SARS-CoV-2, but their efficacy is still controversial 22. Therefore, alternative antiviral agents/therapies are an urgent requirement to stop the spread of the present infection. SARS-CoV-2 enters into host cells mainly through interaction with cell surface receptor ACE2. The most common strategy for current discovery of therapeutics and preventative treatments against SARS-COV-2 is to block the attachment between virus S-protein and ACE2, thus inhibit the invasion of SARS-COV-2 into host cells.

Vaccines targeting the RBD of S-protein elicit antibodies that neutralize SARS-CoV-2 by directly blocking ACE2 binding. Recombinant S-protein vaccines, whole-virus inactivated vaccines, and live-attenuated vaccines also elicit antibodies that interrupt binding to ACE2. Numerous SARS-CoV-2 vaccines are advancing rapidly through clinical trials 23, 24, and several of them have been authorized for clinical usage. However, the potential risk of ADE for monoclonal antibodies and vaccines, where antibody Fc interactions can promote inflammation in respiratory mucosa, is often associated with poorly neutralizing antibodies and has been reported for other respiratory vaccines and in prior studies of MERS and SARS 25. Although therapy using convalescent plasma containing antibodies from recovered COVID-19 patients has revealed no substantial ADE burdens 26, novel mutations of SARS-CoV-2 may lead to neutralization loss and increase the ADE risk.

Despite antibodies and vaccines, small molecules compounds have been identified to block ACE2, including melatonin, mercaptopurine, toremifene and emodin 27, 28. Small molecules exert many key advantages such as lower cost, higher production, better stability, broader distribution, and convenient administration compared with biologics. However, the less precise mechanisms of action and thus the potential side effects increase clinical risk of small-molecule ACE2 blockers 29.

Peptides, a unique class of pharmaceutical compounds occupy an optimal position between small chemical molecules and large biologic proteins, theoretically combines the beneficial characteristics of these two modalities into optimized therapeutics for medical practice. In fact, over 60 peptide drugs have been approved in the United States and other major markets, and peptides continue to enter clinical trials 30. For SARS-CoV virus, the inhibition of S-protein/ACE2 interaction by targeting the S-protein RBD region with peptides appears as a rationale route to block viral entry. In our present study, we designed series of ACE2 protecting peptides and S-protein neutralizing peptides and found two of them, AYp28 and AYn1, exerted potent inhibiting activities on the pseudo-SARS-CoV-2 virus invasion.

Previous studies have identified residues located in N-terminal helix of ACE2 receptors is critical for binding to S-protein of SARS-CoV or SARS-CoV-2 31-33. Several fragments as antiviral peptides have been extracted from hACE2 (such as 21-43, 27-38, 22-44, 22-57 residues in N-terminal helix of ACE2, and 22-44-linker-351-357 residues of ACE2) that exhibit high-binding affinity to SARS-CoV-2 34. A 23-mer peptide SBP1 (IEEQAKTFLDKFNHEAEDLFYQS), corresponding to the 21-43 residues of N-terminal ACE2 α1 helix, can likely stably bind to SARS-CoV-2 RBD with a KD of 47 nM, which is comparable to full length ACE2 binding affinity to SARS-CoV-2 RBD (14.7 nM) 35. However, the activities of SBP1 in cell-based assays *in vitro* and animal studies *in vivo* are not known. In our present study, AYn1, one truncated SBP1 (30-41) peptide, was further modified with six lysine and palmitic acid to increase its solubility and stability. Cell- and non-cell-based assays revealed that AYn1 specifically binds to S-protein but not N-protein of SARS-CoV-2 (**Fig. 2a and 2b**). Pre-incubation of SARS-CoV-2 pseudotype virus with AYn1 inhibited virus entry into ACE2-expressing HEK293T cells. Moreover, AYn1 peptide at inhibiting activity concentrations showed no cytotoxicity, holding the possibility to be developed as safe and potent antiviral therapeutic to prevent or treat COVID-19. Although the efficacy of AYn1 remained unsatisfied in vivo, our present findings prove the concept that ACE2 derived peptides are effective in neutralizing and inhibiting SARS-CoV-2 infection, and therefore could be further developed to prevent or treat COVID-19.

Another alternative approach for blocking SARS-CoV-2/ACE2 attachment is to competitively occupy (block or protect) the virus-binding regions on ACE2. Antibodies blocking ACE2 could be potentially useful on blocking virus entry to cells, however, currently, few studies are addressing this therapeutic method, so further studies, including preclinical and clinical trials, are necessary. Considering the biosafety concern of antibodies and small molecules, peptides have been proposed as promising candidates for this strategy. In our present study, we found ACE2 protecting peptide AYp28, derived from SARS-CoV-2 S-protein RBD, presented much stronger binding affinity to ACE2 (**Fig. 1a**), than its SARS-CoV S-protein RBD-derived analogue AYp26 (**Fig. 1b**). In addition, AYp28 significantly inhibited invasion of pseudo-SARS-CoV-2 virus into HEK293T/hACE2 cells. The incomplete inhibition might be due to the multi-receptor feature of SARS-COV-2 invasion (Wang et al., 2021; Cantuti-Castelvetri et al., 2021; Wei et al., 2020). Much more importantly, different from those antibodies or small molecule inhibitors, AYp28 showed no impacts on ACE2 enzyme activity of converting Ang II into Ang 1-7 (**Fig. 1c**). Moreover, no significant toxicity was observed in peptide-treated cells (**Fig. S5**). Our findings may indicate that SARS-CoV-2 derived ACE2 protecting peptides could serve as safe and effective candidates for SARS-CoV-2 prevention and therapy.

Cocktail therapy has long been clinically practiced for various highly infectious or fatal diseases, such as Ebolavirus 36, hepatitis C virus 37, and Human immunodeficiency virus 38. Recent studies have proposed a cocktail antibody strategy for combating COVID-19, avoiding mutations-induced antibody resistance 11, 39. Much more interestingly, in our present study, the combination of S-protein neutralizing peptide AYn1 and ACE2 protecting peptide AYp28 is effective to inhibit pseudovirus invasion into HEK293T/ACE2 cells, with no cytotoxicity and potential safety concern. Consistently, cocktail therapy with AYn1 and AYp28 inhibited virus invasion in hACE2 transgenic mice without significant side effects. All these findings may indicate a promising approach to treat or prevent COVID-19 by using multi-targeting peptidic cocktail.

Taken together, our findings in this present study may shed light on peptide-based antiviral therapies and their biosafety. Peptide cocktail therapy may serve as warriors, attacking virus using their spears (S-protein neutralizing peptides) and defending using their shields (ACE2 protecting peptides) to reduce cellular virus load by blocking cellular surface receptors and/or neutralizing virus at the stage of virus entry, thereby preventing COVID-19 illness **(Fig. S7)**. Based on this notion, future studies should be performed to analysis the structure (sequence)-activity relationship of peptides and discover much more potent therapeutic candidates. Future works are required to explore the detailed dose-response relationship of peptides either alone or in combination in vivo, and identify the exact dynamic process of peptides prevention and intervention against SARS-CoV-2 infection in mouse model.

## Materials and methods

**Design and synthesis of peptides.** ACE2 protecting peptides (AYp1-28) were designed based on the sequence of S-protein RBD region of SARS-COV-2 or SARS-COV as shown in **Fig. S1a**. S-protein neutralizing peptides (AYn1-14) were designed according to the sequence of hACE2 (**Fig. S1b**). We further modified our peptides with positive net charged poly-lysine (6K) or poly-arginine (6R) to increase the electrostatic attractions between peptides and targeting proteins. Moreover, negative charge poly-aspartic acid (6D) was modified to peptides to increase the peptides stability. Peptides sequences and their target proteins were summarized in **Table 1** and **Table 2**. Peptides synthesis was performed using the Fmoc solid-phase method by ChinaPeptides Co., Ltd. (Shanghai, China) with a purity of over 99% verified by HPLC and mass spectrometry. All peptides were dissolved in deionized water with solubility >5 mg/mL.

**Molecular Docking.** Molecular docking for peptide-protein interactions was computationally simulated by XtalPi AI Research Center (XARC, Beijing, China) using Rosetta FlexPepDock 40. The SARS-CoV-2 S-protein structure (PDBID: 6LZG) and human ACE2 structure (PDBID: 6LZG) were used as initial models for computational simulations. Total 1000 simulations were performed for each peptide and the following indices were obtained for further evaluations of peptide-protein interactions, including the Solvent-accessible surface area buried at the interface in square angstroms (dSASA_int), Binding energy of the interface calculated with cross-interface energy terms (dG_cross), Interface score (I_sc, sum over energy contributed by interface residues of both parteners), Shape complementarity score (sc_value, range 0~1).

**LSPR analysis**. Localized Surface Plasmon Resonance (LSPR) analysis was conducted with OpenSPRTM instrument (Nicoyalife, Canada). The COOH sensor chip was firstly installed on the OpenSPRTM instrument in accordance with the standard procedure. Run the buffer at the maximum flow rate (150 μL/min) and exhaust the bubble after reaching the signal baseline. Slow down the flow rate of buffer solution (PBS, pH 7.4) to 20 µL/min, then load 200 µL EDC/NHS (20 μL /min, 4min) solution to activate COOH sensor chips. The ACE2 (40 µg/ml) and the S-protein (100 nM) were diluted with activation buffer (total 200 µL). The injection port was rinsed with buffer solution and emptied with air. Fill with 200 µL blocking solution (20 µL/min, 4 min), wash the sample ring with buffer solution and empty it with air. Observe baseline for 5 min to ensure stability. Next, the selected peptides were diluted into a series of solutions with different concentration, which were then injected into the chip with the concentration from low to high. The kinetic parameters of the binding reactions were calculated and analyzed by using TraceDrawer software (Ridgeview Instruments AB, Sweden).

**Peptides-proteins binding assay.** ACE2 protecting peptides or S-protein neutralizing peptides dissolved in deionized water (final concentration 0~10 μM) were coated onto ELISA plates and incubated at 4°C for overnight. For the ACE2 or S-protein binding assay, histone-tagged ACE2 (100 ng/mL) or histone-tagged S-protein (10 ng/mL) was incubated with according peptides at 37°C for 2 hours. The bindings of peptide to protein were detected by addition of rabbit anti-His-HRP and incubation at room temperature for 1 hour. The reaction was developed by adding 200 μL substrate solution (KIT001, Sino Biological Inc., Beijing, China) for 15 minutes at 37 °C and was stopped by adding stop solution. The procedure was schematic illustrated as **Fig. S8**. Readings were obtained by SpectroMax® Absorbance Reader CMax Plus (Molecular Devices, USA) at 450 nm. In ACE2 binding assay, paralleled 96-well plate was also coated with SARS-CoV-2 S-protein (10 ng/mL) as positive control. ELISA results are representative of three independent experiments, with each condition duplicated and presented as the mean ± SD of AYp28 or AYp26 peptide binding affinity [OD reading (% over SARS-CoV-2 S-protein)].

**Cells culture.** HEK293T/hACE2 cell line was purchased from Qing Qi Biotechnology Development Co., Ltd. (Blufbio, Shanghai, China). HEK293T/hACE2 cells were cultured in DMEM (Lonza, Switzerland) containing 10% FBS (BI, Israel), 2 mM L-glutamine, 100 IU/mL penicillin and 100 µg/mL streptomycin (Solarbio, China) under standard cell culture condition of 37 ºC and 5% CO_2_.

**Colocalization assay of peptide binding to ACE2 in cells by confocal microscopy.** HEK293T/hACE2 cells were plated in 35-mm confocal chambers at 10^5^/well for 24 hours and incubated with 20 µM AYp28 peptide at 37°C for 2 hours. Cells were then washed twice by PBS and fixed by 4% paraformaldehyde for 20 minutes at room temperature. Triton X-100 (0.5% in PBS) was then added for cell membrane permeabilization. Twenty minutes later, Triton X-100 was removed and 10% goat serum (diluted in PBS) was added for 2-hour blocking. Anti-ACE2 (1:500, sc-390851, Santa Cruz Biotech., USA) or anti-biotin antibody (1:500, sc-57636, Santa Cruz Biotech., USA) was added and incubated overnight at 4 ºC. After washing (5 minutes × 3), Alexa Fluor® 555 goat anti-rabbit IgG (4413S, CST, USA) and Alexa Fluor® 488 goat anti-mouse IgG (4408, CST, USA) secondary antibodies with appropriate concentration was added and incubated in dark at room temperature for 1 hour. Nuclear DNA was stained by 4, 6-diamidino-2-phenylindole (DAPI). Images were taken by confocal microscope (Olympus SpinSR10, Japan).

***In vitro* cytotoxicity assay.** Cytotoxicity of AYn1 and AYp28 peptides on HEK293T/hACE2 cells was determined using MTT Cell Proliferation and Cytotoxicity Assay Kit (M1020, Solarbio Science & Technology Co., Ltd., Beijing, China). Briefly, cells were seeded in 96-well plate (3×10^3^ cells per well) in DMEM supplemented with 10% FBS and incubated for overnight. Cells were then incubated with various concentrations of peptides for 4 to 48 hours at 37°C. MTT (90 μL per well) was added and incubated for 4 hours at 37°C. Then, formazan solution was added (100 μL per well) and further incubated for 10 minutes at room temperature with shaking. OD values at 490 nm were determined and recorded using SpectroMax® Absorbance Reader CMax Plus (Molecular Devices, USA). Cell culture wells without peptides were served as the negative control and wells containing medium only were used as blank control.

**Total RNA extraction and RT-qPCR.** Total RNA of HEK293T/hACE2 cells or hACE2 transgenic mouse tissues were extracted by using TriPure Isolation Reagent (11667165001, Roche, USA) according to the manufacturer's instruction. Real-time RT-qPCR was performed as described previously 41. Extracted RNA was reverse transcribed into cDNA using Prime Script II 1st Strand cDNA synthesis Kit (6210A, Takara Bio Inc., Beijing, China) with T100 PCR system (Bio-Rad, USA). The cDNA was then amplified using specific primers (**Table S1**) for detecting the relative expression levels of functional genes involved in virus invasion, including CAV2, CXADR, EGFR, RAB1B, SLC1A5 and VPS37B, or using primers (**Table S1**) for detecting the expression of pseudovirus.

**ACE2 activity assay.** HEK293T/ACE2 cells were plated in 6-well plates for 20 hours and treated with AYp28 at 0-80 µM for 1 hour, followed by the addition of substrate AngII at 10 nM for 30 minutes. ACE2 activities in the conditioned media were detected using Ang1-7 ELISA kit (DLdevelop, China) according to the manufacturer's instruction.

**Pseudovirus invasion and peptide treatment.** This assay is sensitive and quantitative, and can be conducted in biosafety level-2 (BSL-2) facilities. Pseudovirus containing SARS-CoV-2 S-protein and a defective HIV-1 genome encoding luciferase as a reporter protein was prepared as previously described with minor modification 42. Supernatants containing SARS-CoV-2 pseudovirus were harvested 48 hours post transfection and used for single-cycle invasion of HEK293T/hACE2 cells.

For S-protein neutralizing peptide treatment, pseudovirus solution (10^10^/mL) was pre-incubated with various concentrations (0 to 27.04 µM) of AYn1 at 37°C for 2 hours, followed by further respective incubation with HEK293T/hACE2 cells in 96-well plate at 3×10^4^ cells/well for 48-72 hours at 37°C. For ACE2 protecting peptide treatment, HEK293T/hACE2 cells were plated in 96-well plates at 3×10^4^ cells/well density and incubated with various concentrations of AYp28 or control peptide (0 to 27.04 µM) at 37°C for 2 hours, followed by adding the pseudovirus at 10^10^/mL for 48-72 hours at 37°C. For combination treatment assay, pseudovirus was preincubated with 0 to 27.04 µM of AYn1 for 2 hours at 37°C and then added into HEK293T/hACE2 cells pretreated with 6.76 µM AYp28 for 2 hours, and further incubated for 48-72 hours at 37°C. Luciferase activity was measured using Promega Luciferase Assay System (Promega, USA) according to the manufacturer's instruction.

**Analysis of pseudovirus invasion in HEK293T/hACE2 cells by confocal microscopy.** Confocal microscopy analysis was performed to study the invasion of pseudovirus in HEK293T/hACE2 cells and the inhibition activity of AYn1 and AYp28. Briefly, HEK293T/hACE2 cells (3×10^4^ cells/well) were seeded into 35-mm confocal chambers. Pseudovirus solution (10^10^/mL) was pre-incubated with biotin-labelled AYn1 (60 µM) at 37°C for 2 hours. HEK293T/hACE2 cells were incubated with biotin-labelled AYp28 (20 µM) at 37°C for 2 hours, then were mixed with above mentioned AYn1-neutralized pseudovirus for further 48 hours at 37°C. Pseudovirus and peptides locations were detected using anti-biotin antibody (for peptides, 1:200, sc-57636, Santa Cruz Biotech., USA) and anti-Firefly Luciferase antibody (for pseudovirus, 1:200, bsm-33318M, Bioss, China). Specific signals were visualized using Alexa Fluor®555 goat anti-rabbit IgG (4413S, CST, USA) and Alexa Fluor®488 goat anti-mouse IgG (4408, CST, USA) secondary antibodies. Nuclear DNA was stained by 4,6-diamidino-2-phenylindole (DAPI). Stained sections were observed using a laser scanning confocal microscope (Olympus SpinSR10, Japan).

**Pull down of S-proteins with biotin-AYn1-immobilized beads.** Biotinylated AYn1 peptides (100 μL, 40 μM) in PBST buffer was mixed with Dynabeads M-280 streptavidin (prewashed by PBS) at room temperature for 15 minutes. AYn1 peptide without biotin or PBST buffer was mixed with beads as negative control or blank control. After removing the supernatant, the beads were washed three times with PBST buffer, and 1 μL (0.25 μg/μL) SARS-CoV-2 S-protein was added to the beads. After mixing at room temperature for 20 minutes, the supernatant was collected. The beads were washed three times with PBS. The supernatant and heat elution from the beads were loaded on SDS-PAGE gel and determined using primary antibody (Anti-SARS-S1 rabbit polyclonal antibody, 1:2000, sinobiological, China) and secondary antibody (Goat Anti-rabbit IgG (H+L)/HRP, 1:10000 dilution, Immunoreagents Inc. USA).

**Animals.** K18-hACE2 transgenic mice (TghACE2, 7-8 weeks old, (B6.Cg-Tg(K18-ACE2) 2Prlmn/J (B6J) were purchased from Jackson Laboratories (BarHarbor, ME) and housed in BSL-2 laboratory under standard conditions (22 ~ 25 °C temperature with 40% ~ 60% humidity, 12 hours/12 hours dark/light cycle (light on from 7:00 AM) with standard mice chow and water ad libitum. All experimental protocols were performed according to the Standard Operating Procedures approved by BSL-2 Animal Facilities of Guizhou Medical University. Animals care and procedures were carried out in accordance with guideline approved by the Committee on the Use of Live Animals in Teaching and Research at Guizhou Medical University.

**SARS-CoV-2 Spike-pseudovirus infection and peptides treatment in hACE2 mice.** The animals grouping and treatment were schematically illustrated in **Fig. 4a**. Female hACE2 transgenic mice at 7-8 weeks of age were randomized into 6 groups (3 mice per group). Animals in groups A to C were dripped intranasally with 5 μL AYn1 (2 μg/μL) +5 μL AYp28 (2 μg/μL), 5 μL AYn1 (2 μg/μL) or 5 μL AYp28 (2 μg/μL), respectively. All peptides were labeled by biotin. Three hours after peptides treatment, animals in group A to D were intraperitoneally injected with SARS-CoV-2 Spike-pseudovirus containing firefly luciferase gene (10 μL per mouse, 2×10^7^ copies/mL, SinoBiological, China). At day 2, day 5 and day 10 post the pseudovirus exposure, mice in groups A to E were intraperitoneally injected with luciferin (15 mg/mL, 200 μL per mouse), while animals in group F were injected with normal saline (200 μL per mouse) as blank control. Immediately after luciferin or saline injection, animals were anesthetized by isoflurane and subjected to luciferin imaging using IVIS Lumina III in vivo imaging system (PerkinElmer, USA) to detect the invasion of pseudovirus. The tissues including the lungs, livers, kidneys and small intestines were prepared for immuohistochemical staining or PCR to detect the pseudovirus or peptides locations. The primers sequences used were summarized in **Table S1**.

**Immunohistochemical staining.** Immunohistochemical (IHC) staining was performed on paraffin-embedded 4 μm tissue sections. Briefly, paraffin-embedded sections were baked at 60 °C for 2-4 hours, and deparaffinized with xylene (2 changes × 15 min) and graded ethanol (100% 2 × 5 minutes, 85% 1 × 5 minutes, 75% 1 × 5 minutes). After rehydration, antigen retrieval was performed by boiling the slides in 0.1 M sodium citrate pH 6.0 with microwave. After cooling and rinsing with PBS, quenching of endogenous peroxidase was performed with 3% hydrogen peroxide in methanol for 25 minutes, slides washed in PBS, and blocked with 3% BSA, for 25 minutes in dark at room temperature. Primary antibodies (1:200, anti-biotin (Abcam, USA) for biotin-labeled peptides or anti-luciferase (Bioss, China) for pseudovirus) were diluted and incubated over night at 4 °C. GAPDH was used as control. Secondary antibodies were applied for 50 minutes at room temperature. After washing in PBS, DAB staining was performed.

**Statistical analysis.** The statistical significance between two groups was analyzed by the two-tailed Student *t* test using graphpad prism. Results were considered significant at *P*<0.05.

## Supplementary Material

Supplementary figures and table.

## Figures and Tables

**Table 1:** Molecular docking for ACE2 protecting peptides

	Peptide sequences	Docking protein	dG_cross	dSASA_int	sc_value	I_sc
AYp28	***KKKKKKVEGFNCYFPLQS***	ACE2	-34.4496	1531.23655	0.56795	-33.70445
AYp26	*KKKKKKYKYRYLRHGKLR*	ACE2	-32.54045	1507.7399	0.5771	-31.19635
AYp7	DDDDDDYLYRLF	ACE2	-32.4877	1193.2851	0.65285	-31.74535
AYp5	KKKKKKYLYRLF	ACE2	-32.40755	1338.0122	0.607	-30.8578
AYp27	VEGFNCYFPLQS	ACE2	-31.5179	1121.02775	0.65705	-32.3374
AYp24	KKKKKKYLYRLFRKSNLK	ACE2	-31.07315	1419.467	0.6084	-29.8306
AYp8	KKKKKKYKYRLF	ACE2	-29.72035	1286.8167	0.61665	-28.97565
AYp19	DDDDDDYKYRYL	ACE2	-29.18715	1194.00725	0.59665	-29.30365
AYp21	RRRRRRYKYRYL	ACE2	-28.97495	1373.1271	0.59535	-29.22065
AYp10	KKKKKKRLFRKS	ACE2	-28.70975	1287.17935	0.61905	-28.1619
AYp18	KKKRRRYKYRYL	ACE2	-28.53455	1343.1097	0.60045	-28.51735
AYp22	HHHYKYRYL	ACE2	-27.98075	1258.0382	0.66075	-28.19665
AYp4	KKKYLYRLF	ACE2	-27.1139	1002.9328	0.67	-26.63285
AYp17	KKKKKKYKYRYL	ACE2	-26.4989	1268.80585	0.59355	-26.3129
AYp20	RRRYKYRYL	ACE2	-25.6448	1012.5298	0.65965	-25.30435
AYp25	YKYRYLRHGKLR	ACE2	-25.5586	1121.8961	0.65115	-25.36815
AYp16	KKKYKYRYL	ACE2	-25.5087	1046.48955	0.64195	-25.20135
AYp23	YLYRLFRKSNLK	ACE2	-24.96605	1137.3647	0.633	-25.1026
AYp3	YLYRLF	ACE2	-21.6518	760.0564	0.7118	-21.45285
AYp15	YKYRYL	ACE2	-20.5782	749.1877	0.71115	-20.7058
AYp9	RLFRKS	ACE2	-17.67025	535.34135	0.70165	-16.7909

Solvent-accessible surface area buried at the interface in square angstroms (dSASA_int); Binding energy of the interface calculated with cross-interface energy terms (dG_cross); Interface score (I_sc, sum over energy contributed by interface residues of both parteners); Shape complementarity score (sc_value, range 0~1)

**Table 2:** Molecular docking for S-protein neutralizing peptides

	Peptide sequences	Docking protein	dG_cross	dSASA_int	sc_value	I_sc
AYn1	***KKKKKKDKFNHEAEDLFY***	S-protein RBD	-26.67675	1299.1106	0.5374	-28.0335
AYn7	*KKKKKKDKFNHEAEDLFYMYPLQEIQNLTVGKGDFR*	S-protein RBD	-25.41305	1294.3329	0.54775	-26.4423
AYn14	DKFNHEAEDLFYMYPLQEIQNLTVGKGDFR	S-protein RBD	-25.0505	1176.673	0.5960	-26.5595
AYn8	DKFNHEAEDLFY	S-protein RBD	-25.00335	989.77225	0.5769	-25.6117
AYn13	DKFNHEAEDLFYMYPLQEIQNLTV	S-protein RBD	-22.86635	1234.957	0.571	-24.4427
AYn9	MYPLQEIQNLTV	S-protein RBD	-22.0453	765.0674	0.7005	-22.4894
AYn11	DKFNHEAEDLFYGKGDFR	S-protein RBD	-21.60105	1062.10875	0.651	-23.8136
AYn6	KKKKKKDKFNHEAEDLFYMYPLQEIQNLTV	S-protein RBD	-21.05485	1100.89835	0.5934	-21.7423
AYn4	KKKKKKDKFNHEAEDLFYGKGDFR	S-protein RBD	-19.02995	1141.2023	0.5215	-19.6329
AYn2	KKKKKKMYPLQEIQNLTV	S-protein RBD	-19.0181	935.6129	0.627	-19.6404
AYn5	KKKKKKMYPLQEIQNLTVGKGDFR	S-protein RBD	-18.8448	852.96225	0.6272	-19.4458
AYn12	MYPLQEIQNLTVGKGDFR	S-protein RBD	-17.7617	923.6708	0.5764	-18.6723
AYn10	GKGDFR	S-protein RBD	-15.9727	681.2111	0.5977	-17.1947
AYn3	KKKKKKGKGDFR	S-protein RBD	-14.9821	662.0948	0.6264	-15.9126

Solvent-accessible surface area buried at the interface in square angstroms (dSASA_int); Binding energy of the interface calculated with cross-interface energy terms (dG_cross); Interface score (I_sc, sum over energy contributed by interface residues of both parteners); Shape complementarity score (sc_value, range 0~1)
