# Supplementary material for: Inhibition of SARS-CoV-2 pseudovirus invasion by ACE2 protecting and Spike neutralizing peptides: An alternative approach to COVID19 prevention and therapy
Source: Int J Biol Sci. 2021 Jul 13;17(11):2957–69. doi: 10.7150/ijbs.61476 (PMC8326117; doi:10.7150/ijbs.61476)
Supplement: Supplementary file 1 — Supplementary figures and table. [file ijbsv17p2957s1.pdf]

Supplementary Table 1 RT-qPCR primers

Gene	Primer	Oligonucleotide sequence (5'to 3')
ACE2	ACE2-F	CATTGGAGCAAGTGTTGGATCTT
	ACE2-R	GAGCTAATGCATGCCATTCTCA
CAV2	CAV2-F	TACAAGTTCCTGACGGTGTT
	CAV2-R	AACCATTAGGCAGGTCTTTA
CXADR	CXADR-F	CTCCTGCTGTGCTTCGTG
	CXADR-R	GGCAGATAGGCAGTTTCC
SLC1A5	SLC1A5-F	TTATCCGCTTCTTCAACTCC
	SLC1A5-R	GTAAACCCACATCCTCCATCT
EGFR	EGFR -F	GCCAAGGCACGAGTAACAAG
	EGFR -R	CAATGAGGACATAACCAGCCAC
VPS37	VPS37-F	AGACCCTGTTAGCACTTCTTCA
	VPS37-R	GTGGGCCAGTTTCCGTTT
NUP98	NUP98-F	CCTGACACTTCCCCTTCC
	NUP98-R	GTAAACCTGTCTTATGACCGAAAC
RAB1B	RAB1B -F	CAAGTCATGCCTGCTCCT
	RAB1B -R	CCATCCAGCTCGATGGTTCGGA
IFITM3	IFITM3-F	TCCCTGTTCAACACCCTCTTC
	IFITM3-R	CCAACCATCTTCCTGTCCCTA
TMPRSS2	TMPRSS2-F	GTGCATCACCTTGACCCTG
	TMPRSS2-R	GACCCTATCTCACGCTGAGG
WPRE	WPRE -F	ATTGCCACGGCGGAACTC
	WPRE -R	AGCAGCCAAGGAAAGGACGA
IL-6	IL-6-F	CTCCCAACAGACCTGTCTATAC
	IL-6-R	CCATTGCACAACTCTTTTCTCA
GAPDH	GAPDH-F	AGGTCGGTGTGAACGGATTTG
	GAPDH-R	TGTAGACCATGTAGTTGAGGTCA
CXCL10	CXCL10-F	CCAAGTGCTGCCGTCATTTTC
	CXCL10-R	GGCTCGCAGGGATGATTTCAA
CCL5	CCL5-F	GCTGCTTTGCCTACCTCTCC
	CCL5-R	TCGAGTGACAAACACGACTGC
CXCL11	CXCL11-F	GGCTTCCTTATGTTCAAACAGGG
	CXCL11-R	GCCGTTACTCGGGTAAATTACA
Ifnb	Ifnb -F	CAGCTCCAAGAAAGGACGAAC
	Ifnb -R	GGCAGTGTAACTCTTCTGCAT
CCL2	CCL2-F	AGGTCCCTATGGTGCCAATGT
	CCL2-R	CGGCAGGATTTTGAGGTCCA
IFN-γ	IFN-γ-F	TACCAGAACATGTCACAGACTC
	IFN-γ-R	ATGATCAGAAATGTTGGTGCAG

Supplementary Figures

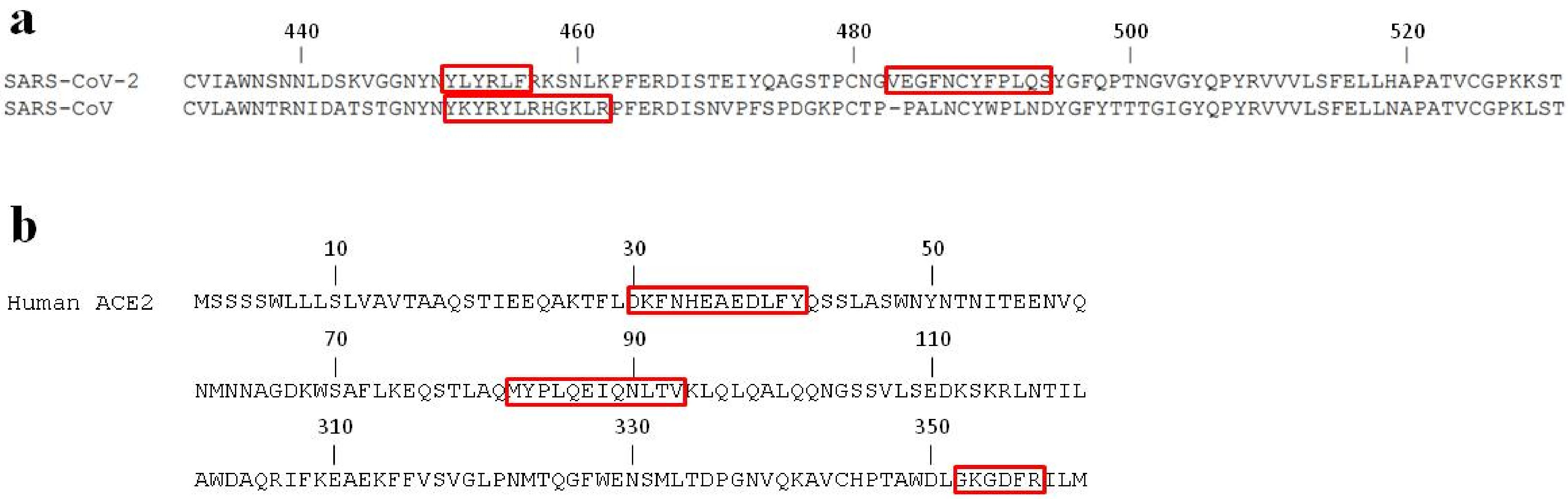

Supplementary Fig. S1 Design of S-protein neutralizing peptides and ACE2-protecting peptides. ACE2 protecting peptides (AYp1-28) were designed and modified based on the sequence of S-protein RBD region of SARS-COV-2 or SARS-COV (**a**). S-protein neutralizing peptides (AYn1-14) were designed and modified according to the sequence of hACE2 (**b**).

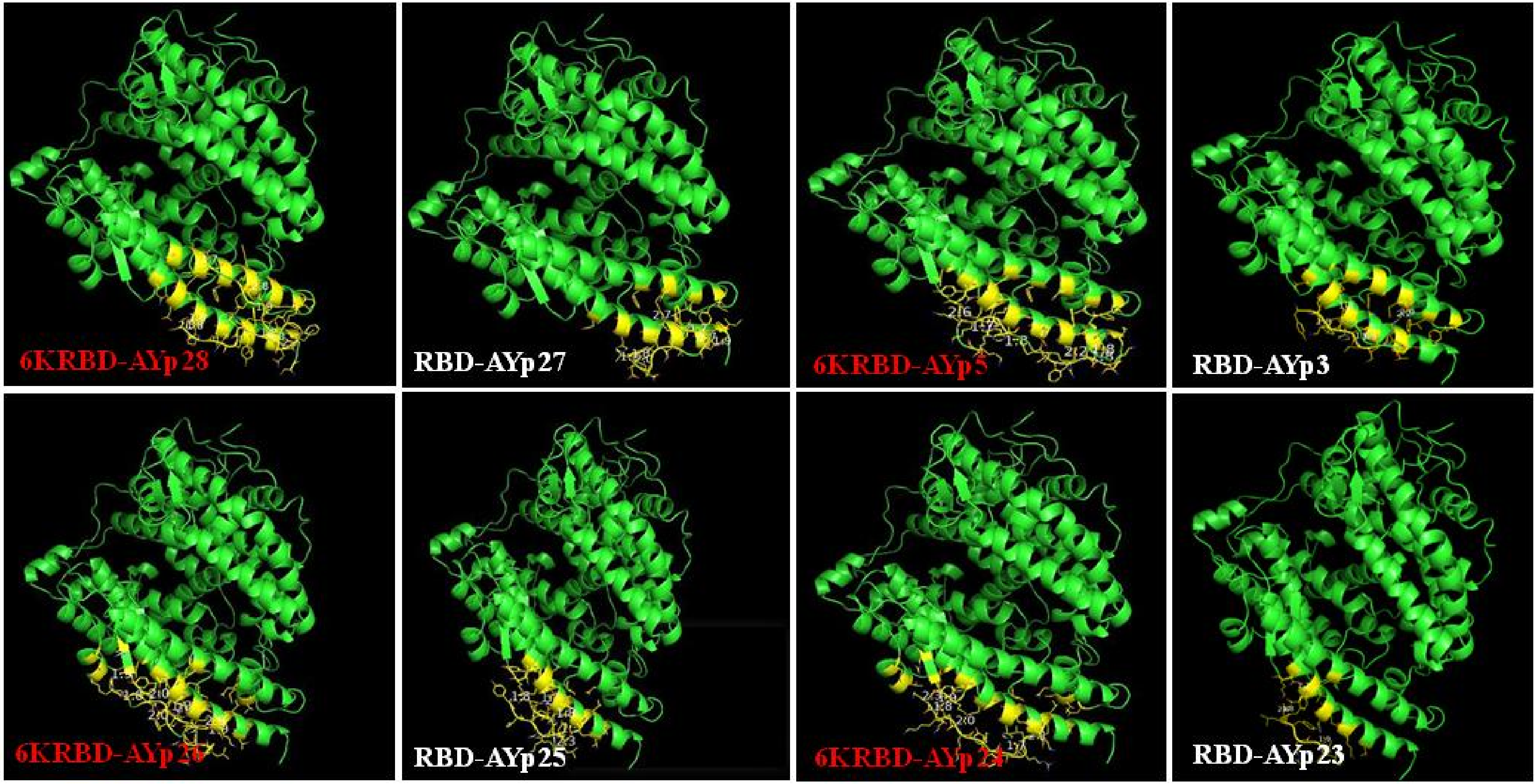

Supplementary Fig. S2 Computational simulation of interactions between ACE2-protecting peptides and ACE2 protein. Among 21 tested ACE2 protecting peptides, AYp28 showed a larger attachment surface with ACE2 protein (yellow area).

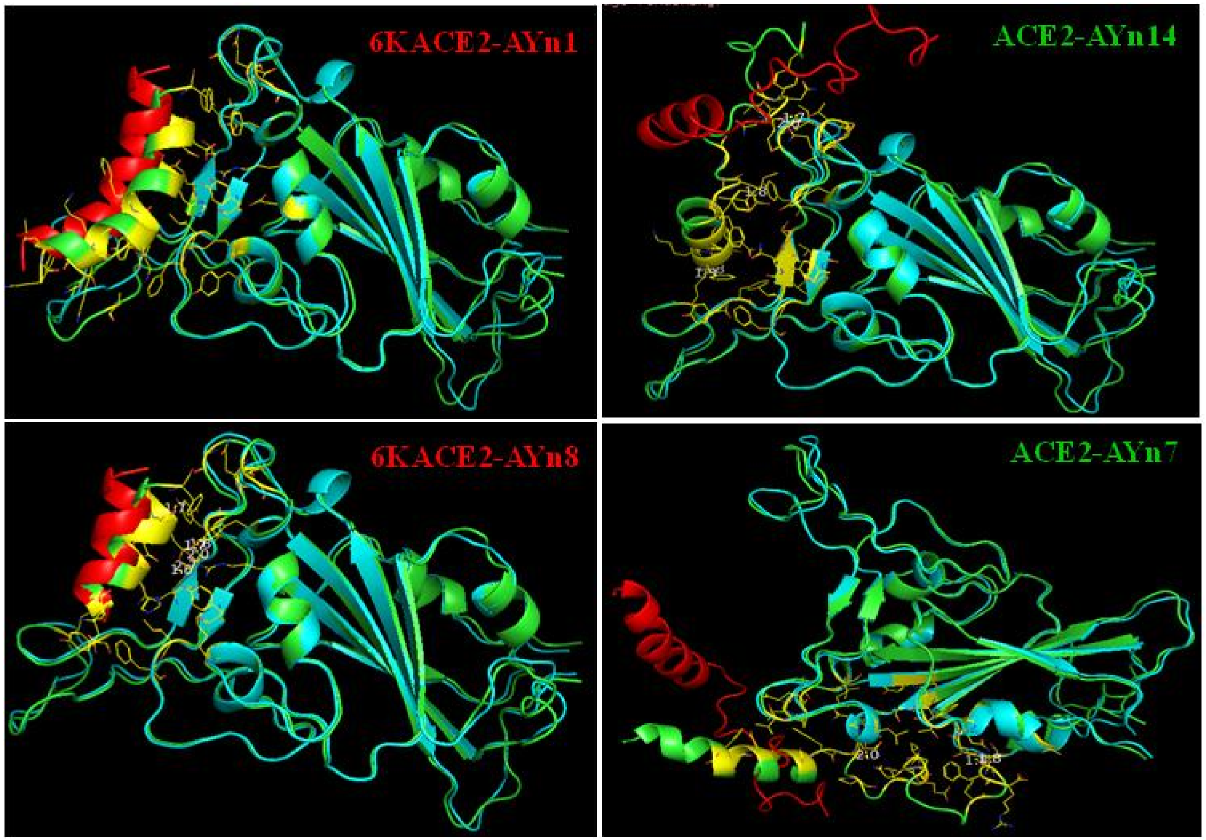

Supplementary Fig. S3 Computational simulation of interactions between S-protein-neutralizing peptides and S-protein. Among 14 tested S-protein neutralizing peptides, AYn1 presented the largest binding surface (yellow area).

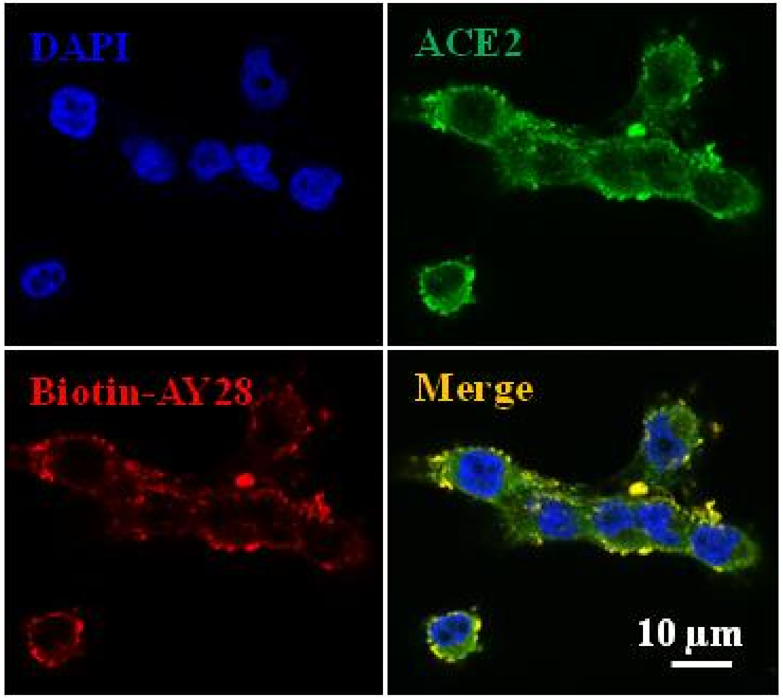

Supplementary Fig. S4 Interaction of AYp28 with ACE2 protein as assessed by confocal microscopy. Biotin-labeled AYp28 peptide was incubated with HEK293T/hAEC2 cells and observed by confocal microscopy. Clear colocalizations of AYp28 (red) with cell membrane ACE2 (green) were found.

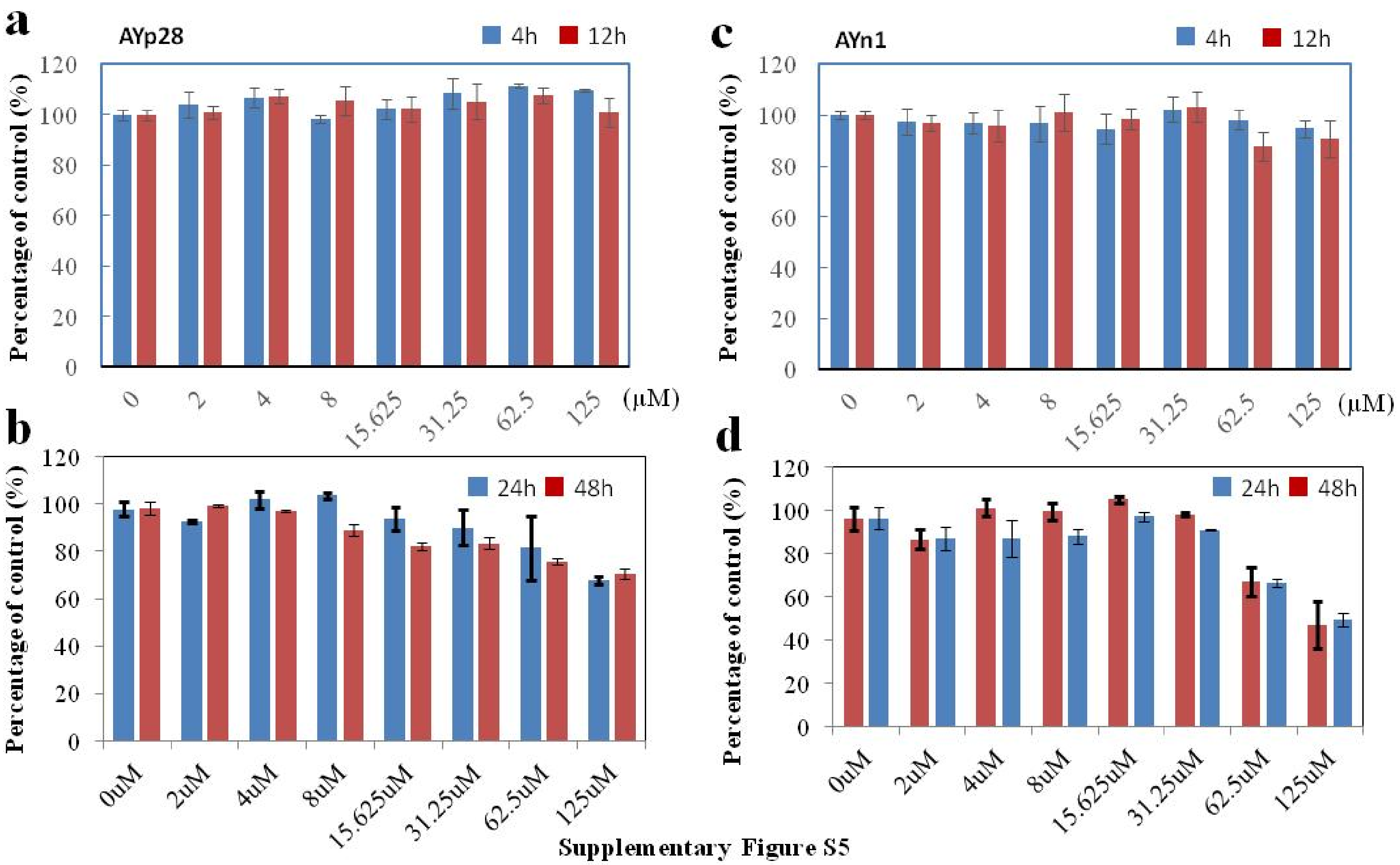

Supplementary Fig. S5 Cytotoxicity of AYp28 and AYn1 in HEK293T/hACE2 cells as assessed by MTT assay.

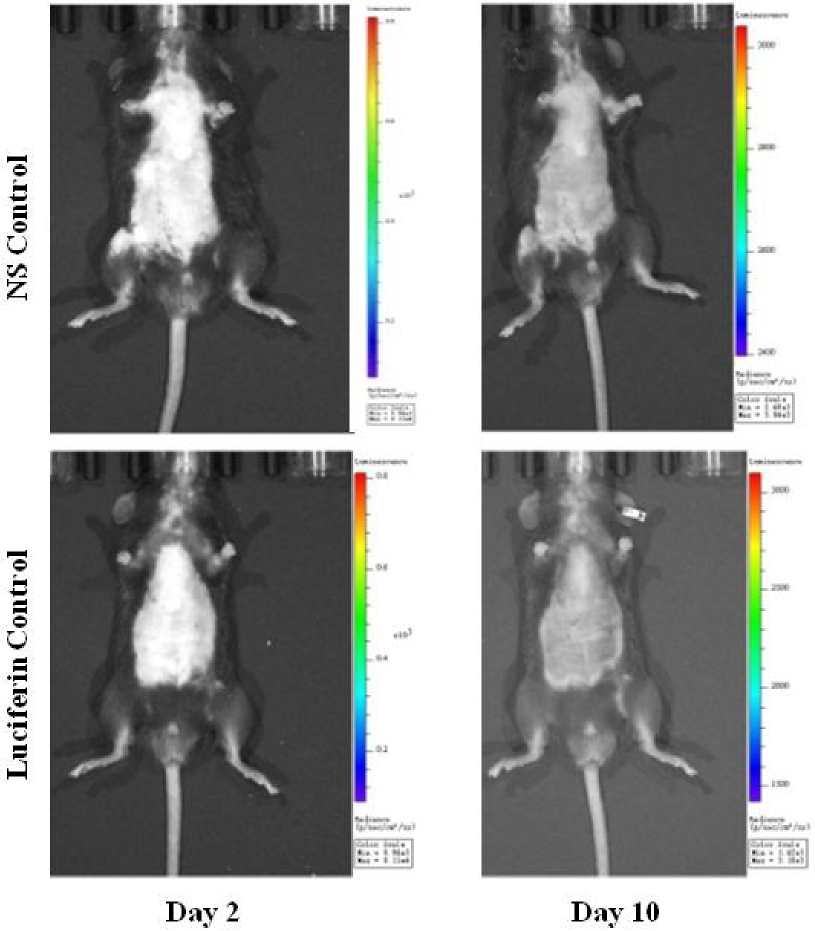

Supplementary Fig. S6 Luciferin fluorescence signal observed in PBS and luciferin control mice.

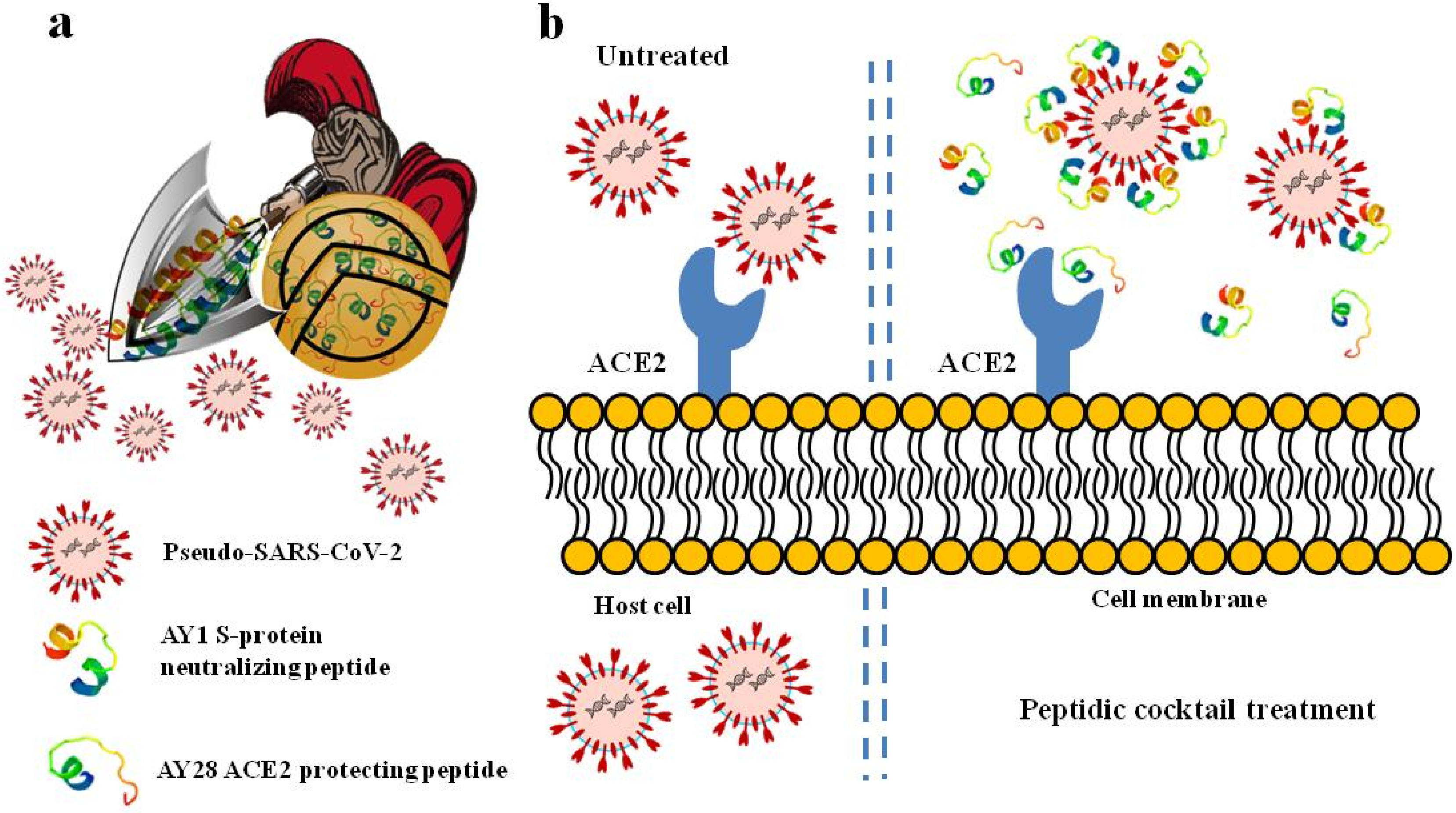

Supplementary Fig. S7 Schematic illustrations of the mechanisms synergistic activity of ACE2-protecting and S-protein neutralizing peptidic cocktail against SARS-CoV-2.

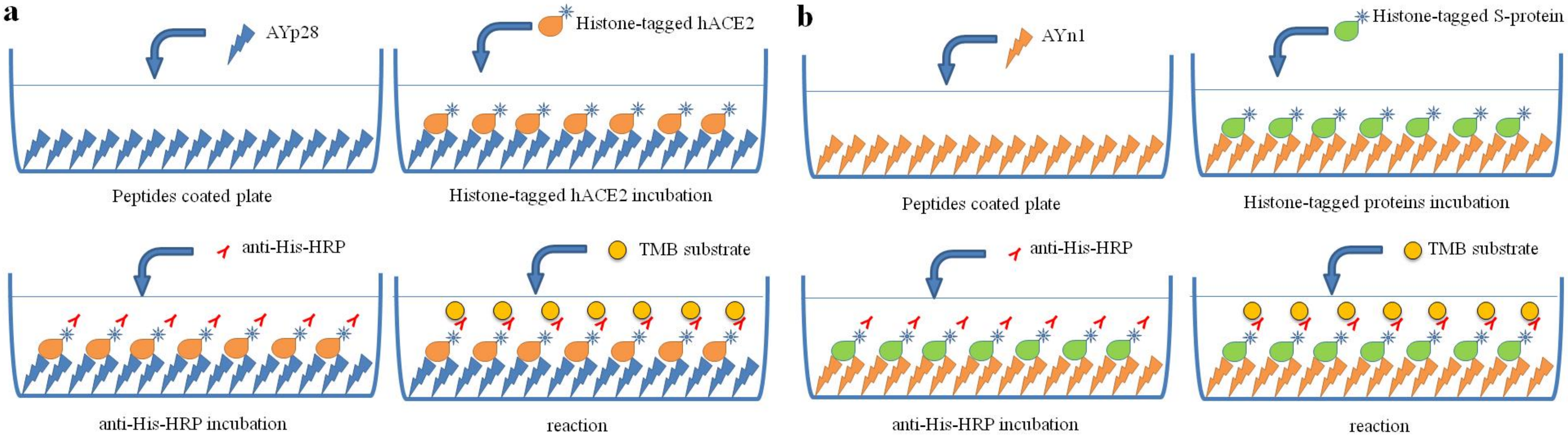

Supplementary Fig. S8 Schematic illustration of experimental protocol of ELISA-like peptides-proteins binding assay. ACE2 protecting peptides or S-protein neutralizing peptides dissolved in deionized water were coated onto ELISA plates and incubated at 4°C for overnight. For the ACE2 (**a**) or S-protein binding assay (**b**), histone-tagged ACE2 (100 ng/mL) or histone-tagged S-protein (10 ng/mL) was incubated with according peptides at 37°C for 2 hours. The bindings of peptide to protein were detected by addition of rabbit anti-His-HRP and incubation at room temperature for 1 hour. The reaction was developed by adding 200 μL substrate solution for 15 minutes at 37 °C and was stopped by adding stop solution.
